# Active unicameral bone cysts: control firstly, cure secondly

**DOI:** 10.1186/s13018-019-1326-3

**Published:** 2019-08-28

**Authors:** Qing Liu, Hongbo He, Hao Zeng, Yuhao Yuan, Zhiwei Wang, Xiaopeng Tong, Wei Luo

**Affiliations:** 1 Department of Orthopaedics, Xiangya Hospital, Central South University, Changsha, 410008 China; 2 Department of Spine Surgery, The Second Xiangya Hospital, Central South University, Changsha, China

**Keywords:** Unicameral bone cysts, Percutaneous curettage, Autogenous bone marrow grafting, Intracystic methylprednisolone injection

## Abstract

**Purpose:**

This retrospective study evaluated the efficacy of minimally invasive surgery to control cyst progression for active unicameral bone cysts (AUBC) by intracystic methylprednisolone injection, percutaneous curettage, and autogenous bone marrow grafting.

**Methods:**

From May 2010 to May 2017, patients diagnosed with AUBC who underwent percutaneous double-needle intracystic methylprednisolone injection, percutaneous curettage, and autogenous bone marrow grafting were retrospectively reviewed. Recurrence was defined by modified Neer scale score. Patients were followed up regularly, and previous imaging findings were compared to evaluate treatment efficacy.

**Results:**

The 26 patients (17 boys, 9 girls, mean age, 9.4 ± 3.1 years) were followed up for a mean 45.1 months (range, 24–82 months). Follow-up consisted of clinical evaluation and radiographic review. Twenty patients (77%) achieved latent disease stage after the first treatment, while six (23%) achieved it after the second treatment. Postoperative pathological fracture imaging scores were score I in 18 (70%), score II in five (19%), score III in two (8%), and score IV in one patient (4%). All 26 patients returned to their full activities and were asymptomatic at the most recent follow-up. The success rate (scores I and II) independent of the number of treatments was 89%. Treatment time was correlated with cyst size and length. Sex, age, cyst location and size, pathological fracture, and other clinical factors or radiological data did not influence the curative effect. No other complications occurred.

**Conclusions:**

For AUBC, minimally invasive treatment is feasible to control cyst progression and then cure it without sequelae. Intracystic methylprednisolone injection, percutaneous curettage, and autogenous bone marrow grafting are an excellent choice.

## Introduction

Unicameral bone cysts (UBC), also known as simple or solitary bone cysts, are the only genuine cysts of the bone. During childhood, about 15% of simple bone cysts heal without treatment, but most persist or enlarge [1]. UBC commonly occurs in the metaphyseal-diaphyseal region of long bones, particularly the humerus and femur [2], in the first two decades of life, with a peak at the age of 10 [3–5]. UBC are frequently asymptomatic and may regress spontaneously [4, 6], but they are typically discovered incidentally or after a fracture.

**Fig. 1:**
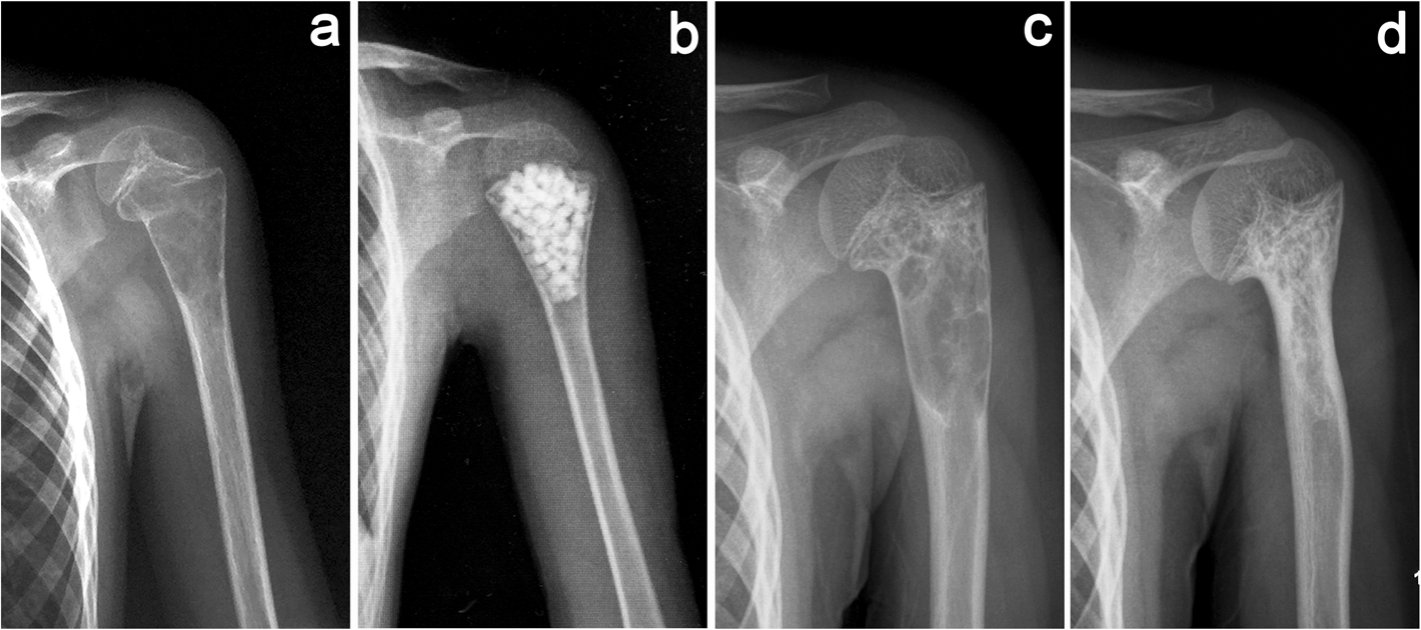
Limb deformities caused by focal curettage and bone grafting to treat an active unicameral bone cyst (AUBC). **a** A 5-year-old boy with a left proximal humerus AUBC and a pathological fracture. **b** Curettage and bone grafting were performed, and forearm suspension was limited for 2 months postoperative. **c** Partial healing of the cyst with slight deformity 3 months postoperative. **d** The cyst healed completely, and the proximal humerus showed obvious deformities at 9 months postoperative

UBC are usually classified into active (juxtaposed with epiphyseal growth plate and at a distance < 0.5 cm [7, 8]) or latent (migrating away and separating from the growth plate by a normal area of cancellous bone) type. The long axis of active unicameral bone cysts (AUBC) is consistent with the longitudinal axis of the bone shaft; conversely, its transverse diameter is often narrower than that of the epiphyseal plate, making it unlikely for them to regress and prone to damage the epiphysis, resulting in a higher risk of fracture and the risk of skeletal deformity during curettage [9, 10](Fig. 1).

It is accepted that the more radical the cyst excision, the lower the rate of recurrence [11]. However, UBC as benign self-limiting lesion does not require radical surgery, especially with the high rate of associated complications including injury to the growth plate for AUBC. A number of secure, minimally invasive, and convenient methods [12] have recently been proposed to treat UBC. Repeated percutaneous injection of corticosteroids, proposed by Scaglietti in 1979 [13], has been widely used due to its simplicity and high healing rate. With the development of medical technology, percutaneous aspiration and autogenous bone marrow injections [3, 12, 14–16] have also been widely advocated for being more effective and reliable than a single injection. Injection of bone marrow alone [14, 17, 18] or in combination with a demineralized bone matrix [8] has also been proposed as an alternative to steroids for treating UBC.

There are very few studies on AUBC, and controversy persists regarding its standard treatment. Therefore, how should we choose the treatment for AUBC? The fracture risk of the lesion site and protecting the epiphysis should be considered in the treatment. Rougraff and Kling [8] reported treating AUBC with a percutaneous injection of demineralized bone matrix combined with autogenous bone marrow in 2002. The results showed that only 16 (70%) patients were healed after treatment. Ahn proposed that AUBC in the upper limb are at greater risk of fracture [19]. More recent studies have suggested that AUBC treatment outcomes may be significantly worse than those of latent UBC; in as many as half of the patients, the lesion relapsed. These unsatisfactory results prompted a search for other treatments that can stimulate osteogenesis and facilitate the AUBC healing process.

This study evaluated the clinical outcomes of AUBC treatment consisting of intracystic methylprednisolone injection, percutaneous curettage, and autogenous bone marrow grafting. The effectiveness of this method was studied according to different factors that might affect the outcome including cyst aggression. The patients and their families were informed of the risks and potential benefits of the study and provided consent to participate.

## Methods

### Patients

From May 2010 to May 2017, 113 consecutive patients with a diagnosis of UBC were treated at the Xiangya Hospital Bone Tumor Center. The inclusion criteria were diagnosis of AUBC; received treatment consisting of intracystic methylprednisolone injection, percutaneous curettage, and autogenous bone marrow grafting; and completed long-term follow-up. The exclusion criteria were latent UBC, treatment with other means, and loss to follow-up. A total of 26 patients met the criteria and were included in the study. The diagnosis was based on typical imaging (X-ray showing osteolytic lesions located at the epiphysis of the long bone with smooth edges and no periosteal reaction, computed tomography value the same as the density of water, enhanced magnetic resonance imaging showing marginal enhancement of the lesion), clinical manifestations (symptoms are more insidious; most patients suffer from pain, swelling, and dysfunction due to pathological fractures), and postoperative histopathology (histopathologically confirmed UBC postoperatively). Age, sex, cyst location, cyst length and size, history of previous fracture, follow-up duration, and number of treatments were recorded (Table 1). Cyst size was defined as the ratio of the cyst length to the adjacent epiphysis length [12], a value that can be compared directly without requiring correction for age and avoids errors resulting from radiological magnification. This study was approved by the Research Ethics Committee of Xiangya Hospital. The patients and their families were invited to participate and provided informed consent upon learning of the study’s risks and benefits.

**Table 1:** Patients’ demographic and surgical data

Patients number	Cyst length (cm)	Epiphyseal length (cm)	Cyst size*	Cyst location	Prior fracture	Times of treatment	Transform latent after one treatment	Duration of follow-up (month)	Curative effect	Complication
1	5.8	3	1.9	Proximal humerus	No	1	No	25	Failure	Fracture
2	8.3	5.7	1.5	Distal femur	No	1	Yes	40	Healed	None
3	8.2	5.2	1.6	Proximal tibia	No	1	Yes	51	Healed with defects	None
4	6.3	3.2	2.0	Proximal femur	No	1	Yes	33	Healed	None
5	7.5	5.3	1.4	Proximal tibia	No	1	Yes	76	Healed	None
6	7.08	4.8	1.5	Distal femur	No	2	No	65	Healed	None
7	5.9	3.9	1.5	Proximal femur	No	1	Yes	45	Healed	None
8	5.6	2.6	2.2	Proximal humerus	No	2	No	27	Healed	None
9	2.6	1.8	1.4	Proximal femur	Yes	1	Yes	30	Healed	None
10	3.8	2.4	1.6	Proximal humerus	Yes	1	Yes	26	Healed with defects	None
11	4.5	2.1	2.1	Proximal humerus	No	2	No	31	Healed with defects	None
12	4.6	2.7	1.7	Proximal tibia	No	1	Yes	26	Healed	None
13	4.1	3.4	1.2	Proximal humerus	No	1	Yes	78	Healed	None
14	5.5	2.7	2.0	Proximal femur	No	1	Yes	34	Healed	None
15	4.8	2	2.4	Proximal humerus	No	1	Yes	52	Healed	None
16	9.9	3.8	2.6	Proximal humerus	No	1	Yes	80	Healed with defects	None
17	4.7	3	1.6	Proximal femur	No	1	Yes	29	Healed	None
18	4.2	3.2	1.3	Proximal humerus	Yes	1	Yes	26	Healed	None
19	5.3	3.2	1.7	Proximal humerus	Yes	2	No	82	Healed with defects	None
20	9.9	3.8	2.6	Proximal humerus	Yes	1	Yes	58	Healed	None
21	5.2	3.9	1.3	Proximal humerus	No	2	Yes	36	Persistent cyst	None
22	4.9	3.1	1.6	Proximal humerus	Yes	1	Yes	37	Healed	None
23	4.7	3.3	1.4	Proximal femur	Yes	1	Yes	76	Healed	None
24	6.2	1.8	3.4	Proximal tibia	No	1	No	49	Healed	None
25	5.4	3.3	1.6	Proximal humerus	Yes	2	Yes	24	Persistent cyst	None
26	4.8	3.2	1.5	Proximal humerus	No	1	Yes	36	Healed	None

*Cyst size was defined as the ratio of the cyst length to the adjacent epiphysis length

### Surgical technique

**Fig. 2:**
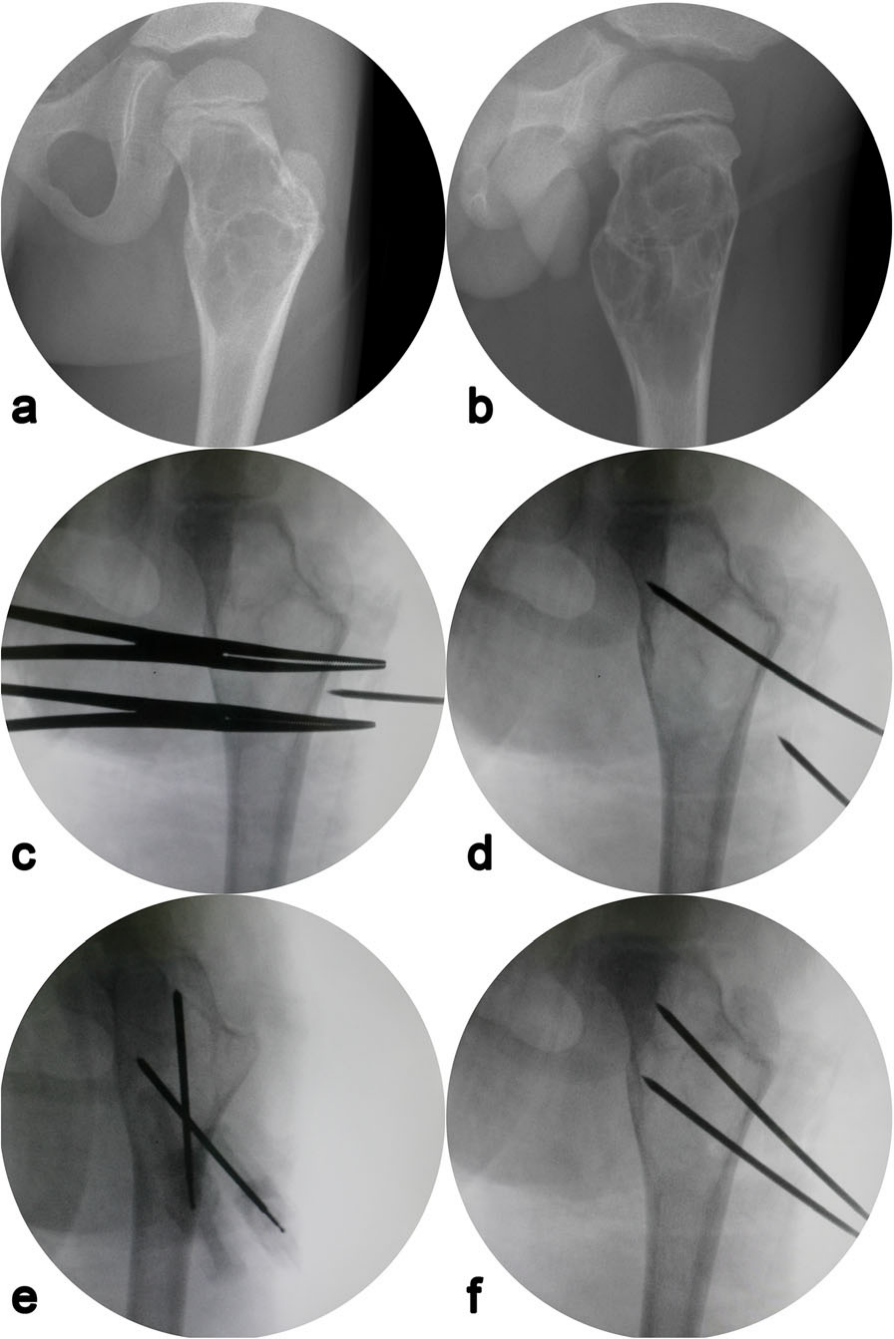
Radiographs of a 6-year-old boy during the operation. **a**, **b** Anteroposterior and lateral radiology manifestation of the cyst before puncture. **c**–**f** Under fluoroscopic guidance, two puncture needles were used to puncture 1/3 to 2/3 of the length of the cyst successively

**Fig. 3:**
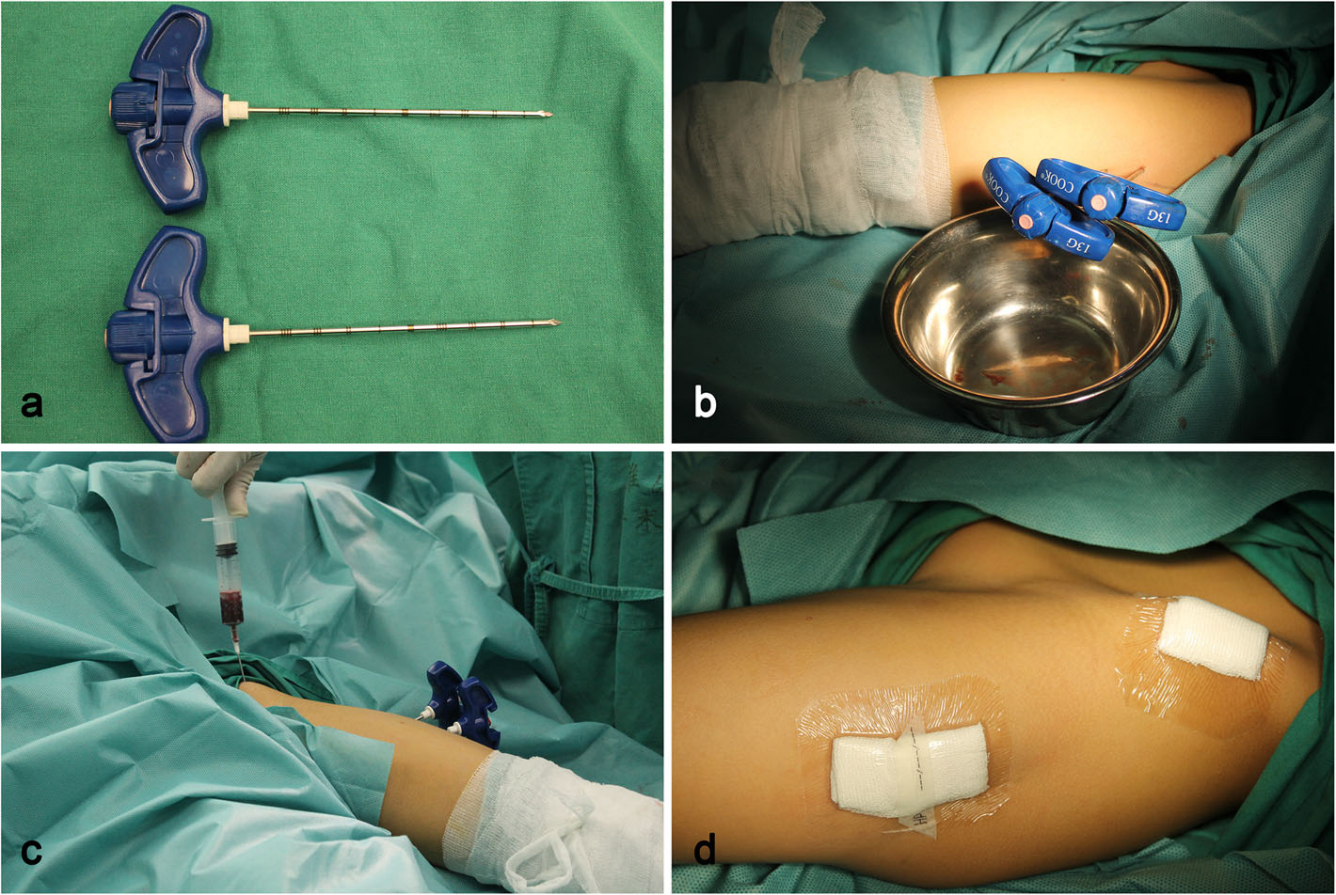
**a** Two 13-gauge/10-cm bone biopsy needles were used. **b** Two puncture needles were inserted into the cyst under the fluoroscopic guidance. **c** Autogenous bone marrow was extracted from the anterior iliac wing. **d** Sticking the puncture point with an ordinary semipermeable membrane

All procedures were performed under intravenous general anesthesia without the use of a tourniquet. Using preoperative enhanced magnetic resonance imaging (MRI), we can judge whether the cystic cavity is unilocular or multilocular and then mark the needle insertion point by a C-arm machine before disinfection (Fig. 2a, b). The puncture location was parallel to the point of 1/3 and 2/3 of the long axis of the lesion. The puncture needles were 13-gauge/10-cm bone biopsy needle (Cook Medical, Bloomington, USA; Fig. 3a). First, two needles were inserted under fluoroscopic guidance from the thinnest part of the cyst wall according to the previous markings (Fig. 2c–f), potentially causing a sense of breakthrough. The needle tip was used to break through the atrial septum of the cystic cavity to change the multilocular structure to a unilocular structure. Second, the cavity was repeatedly perfused with sterile water for injection. Methylprednisolone was injected from the upper pinhole, and pressure was applied to the cyst for 15 min to prevent overflow. The doses of methylprednisolone according to each patient’s body weight were 40–120 mg. Sterile water was then injected two or three more times to reduce the residue of methylprednisolone. Third, we scraped the wall of the capsule as thoroughly as possible using a small spoon or crooked Kirschner needle and gently scratched a portion of the wall close to the epiphyseal plate. We then repeated the previous lavage operation to flush out the cystic wall tissue and send it for pathological examination. And finally, we extracted the autologous red bone marrow from the anterior iliac wing of the patient and slowly injected it into the cavity (Fig. 3c). The amount of red bone marrow was 10–20 mL according to cyst size. The two puncture needles were pulled out, local compression was applied for 5 min, and the injection sites were bandaged (Fig. 3d). A forearm sling or weight-free brace was used for 2–4 weeks postoperative.

### Follow-up and evaluation

Patients were followed up radiographically every 3 months for the first 2 years, every 6 months for the next 2 years, and annually thereafter. A juxtaposition of the mean value of the collected data was performed, while multiple logistic regression analysis was used to evaluate the prognostic significance of age, sex, cyst size, cyst location, and history of previous fracture. Since there is no unified standard to evaluate UBC treatment outcomes, this study evaluated the radiographic films using the modified Neer scale system [20] proposed by Chang CH et al. [12, 21] (Table 2). A cyst that healed well or healed with defects was not considered to require treatment, whereas a persistent cyst or recurrence indicated therapeutic failure for which supplementary treatments were needed.

**Table 2:** Modified Neer classification of radiologic results

Score	Classification	Description	Treatment
I	Healed	Cyst filled with new bone, with or without small radiolucent area(s) < 1 cm in size	Not necessary
II	Healed with defects	Radiolucent area(s) < 50% of the diameter of the bone with enough cortical thickness to prevent fracture	Not necessary
III	Persistent cyst	Radiolucent area > 50% of the diameter of the bone and with a thin cortical rim; no increase of the size of the cyst	Continued restriction of activity, possible repeated treatment required
IV*	Recurrent or nonresponsive cyst	Cyst reappeared in a previously obliterated area or a radiolucent area has increased in size	Need for repeated treatment

*Postoperative pathological fracture belong to score IV

To determine the relationship between different variables and identify the significant factors influencing the curative effect, the collected data were statistically analyzed using SPSS version 20 (SPSS Inc., Chicago, IL, USA). Multiple regression analysis was used to evaluate the prognostic significance of multiple factors. The chi-square test and Fisher’s exact test were also used. Values of *P* < 0.05 were considered statistically significant.

## Results

All cases in this study had confirmed UBC according to intraoperative findings (aspirated bright yellow liquid) and postoperative histopathological examination. No operative complications related to the procedure occurred, and treatment failed in only one patient because of postoperative pathological fracture.

The 17 boys and nine girls had a mean age of 9.4 years (range, 5–16 years) at the first treatment (Table 3). The anatomic sites of the cyst were located in the upper limbs in 14 cases and in the lower extremities in 12 cases. Cyst length was 2.6–9.9 cm (mean, 5.8 cm), and size was 1.2–3.4 cm (mean, 1.8 cm). Eight patients (31%) had pathological fractures at the time of consultation. They were treated with plaster external fixation for 1–3 months until the fracture healed.

**Table 3:** Demographic data for patients with AUBC

Gender*	
M	17 (65%)
F	9 (35%)
Age*	9.4 ± 3.0
Cyst length (cm)*	5.8 ± 1.8 (*P* = 0.032)
Epiphyseal length (cm)*	3.3 ± 1.0
The size of the cyst*	1.8 ± 0.5 (*P* = 0.044)
Cyst location	
Proximal humerus	14 (54%)
Distal femur	2 (8%)
Proximal tibia	4 (15%)
Proximal femur	6 (23%)
Prior fracture*	8 (31%)
Duration of follow-up (months)	45.1 ± 19.7
Curative effect	
Healed	18 (70%)
Healed with defects	5 (19%)
Persistent cyst	2(8%)
Treatment times**	
One	20 (77%)
Two	6(23%)

*Gender, age, location and size of cyst, and pathological fracture had no correlation with the curative effect

**Treatment times had a significant correlation with the length of the cyst (*P* = 0.032) and the size of the cyst (*P* = 0.044)

**Fig. 4:**
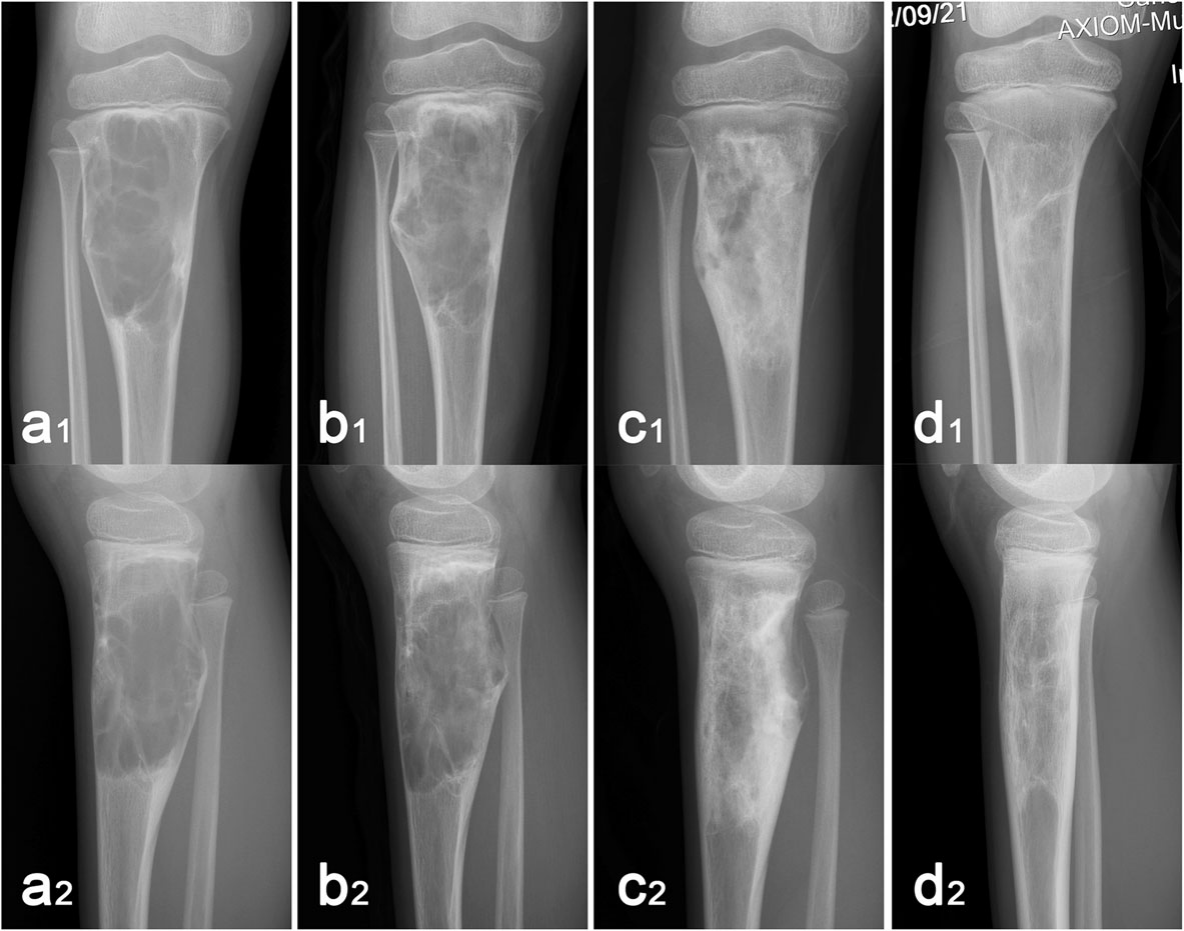
A 10-year-old boy treated with Intracystic methylprednisolone injection, percutaneous curettage, and autogenous bone marrow grafting. These radiographs show changes in the cyst after one treatment as follow-up time prolongated (1, frontal X-ray; 2, lateral X-ray). **a** Anteroposterior and lateral X-ray before the treatment. **b** X-ray at 3 months after the treatment. **c** X-ray at 6 months after the treatment. **d** X-ray at 12 months after the treatment. In our series of follow-up results, we found that the cortical bone of the cyst was gradually thickened; the low-density areas within the cyst ossified gradually and eventually reassumed a normal bone morphology

**Fig. 5:**
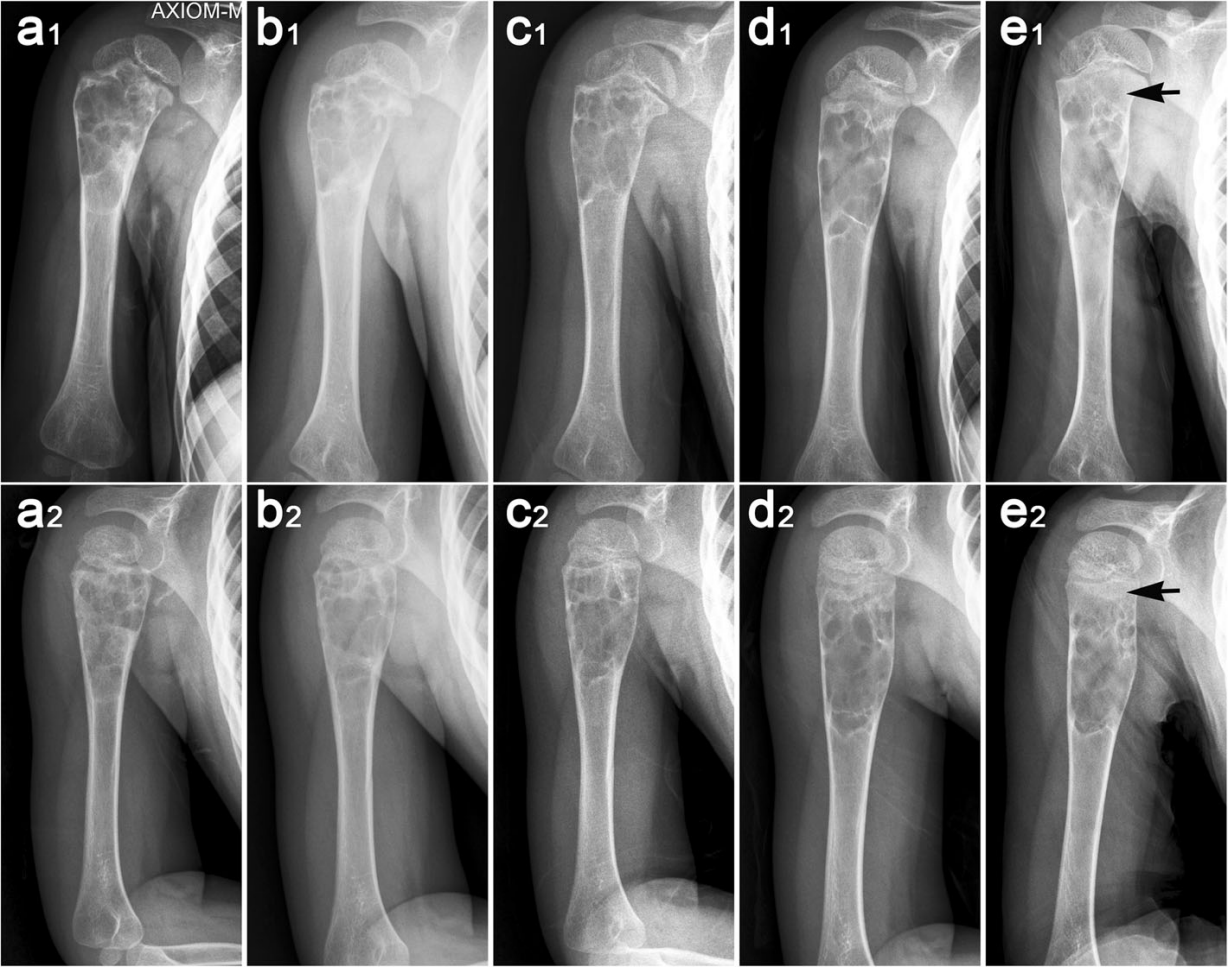
X-ray films of the persistent cysts in a 10-year-old boy after two treatments (1, frontal X-ray; 2, lateral X-ray). **a** An active unicameral bone cyst was found at the proximal humerus due to pathological fracture and treated conservatively for 6 weeks. **b** Two months after the first treatment, no significant change was noted in the cyst. **c** After 2 months of observation, the cyst remained unchanged and was treated for the second time. **d** Three months after the second treatment, the cyst was partially healed and began to move away from the epiphyseal growth plate. **e** Nine months after the second treatment, the radiolucent area filled > 50% of the diameter of the bone and the cyst persisted

Twenty patients (77%) achieved the latent disease stage after the first treatment, while six patients achieved the latent stage after the second treatment. Modified Neer classification of radiologic results [12, 21] revealed that score I was achieved in 18 cases (70%) (Fig. 4), score II in five cases (19%), and score III in two cases (8%) (Fig. 5). Score IV was observed in one patient (4%) as a postoperative pathological fracture; prior to that, the cyst had become latent. The patient healed completely when subjected to extensive curettage and subsequent allograft bone transplantation. All 26 patients returned to full activities and were asymptomatic at the most recent follow-up. The success rate (scores I and II) independent of the number of treatments was 89%. The curative effect was not significantly correlated with the clinical and radiological data, although healing with defects occurred more commonly in cases affecting the proximal humerus.

There was a significant correlation between the number of treatments and cyst length (*P* = 0.032) or cyst size (*P* = 0.044); however, number of treatments had no significant influence on curative effect (*P* = 0.609). Cases of AUBC are more likely to develop pathological fractures than those in the latent stage [7, 20]. The incidence of pathological fracture pre-hospitalization was 31% in this group, consistent with the previous research.

## Discussion

According to biological behavior, UBC is usually classified as active [7, 8] or latent. Why some UBC continue to grow and others remain stationary or even self-heal remains unknown [8]. The purpose and method of the surgical treatment of UBC should be tailored to a cyst’s specific biological behaviors. Nonsurgical treatment is recommended when UBC are discovered incidentally in asymptomatic patients, and no substantial decrease in the strength of the affected bone is observed to warrant invasive intervention. Surgery was needed in the other cases.

Open surgery with radical cyst resection and bone grafting can definitely achieve complete UBC cure [22]. However, excessive surgical trauma and relatively high complication rates reduce its value in the treatment of these self-limited diseases. The etiology of UBC remains obscure. The literature reports that UBC development may be related to epiphyseal plate malformation [23], bone resorption [24], exudate retention [25], and venous obstruction [26]. Moreover, venous obstruction may play an important role in the pathogenesis of UBC. Minimally invasive treatment is aimed at its etiology [2], including reducing pressure, filling the bone defect cavity, curettage of the cyst membrane, breaking the balance of the intra-cyst environment, and promoting osteogenesis. The main current treatment methods for UBC are observation, steroid injection [8], autologous bone marrow injection [27], curettage and bone grafting [28], demineralized bone matrix and autogenous bone marrow injection [21, 29], and intramedullary drilling with or without nailing [30]. Although these treatments are effective [2, 7, 15, 31–33], the recurrence rates were still relatively high in the AUBC group. Therefore, there is no universally accepted standardized treatment for AUBC. A safer, more effective, and minimally invasive treatment for AUBC that feature fewer deficiencies is sought.

Based on previous studies, we also explored a more beneficial surgical approach for patients with AUBC. This method involves intramedullary decompression, followed by intramedullary injection of methylprednisolone, percutaneous curettage of the cyst membrane that slightly destroyed part of the cyst membrane near the epiphyseal plate, and autogenous bone marrow transplantation to treat AUBC close to the epiphyseal plate. Twenty patients achieved latent disease stage after one operation, while six cases required a second operation before achieving the latent stage. The success ratio is far higher than reported by Di et al. [21]. This operation had four novel aspects: First, the cystic cavity was treated with methylprednisolone after decompression and the cyst membrane was scratched extensively. Second, the cyst membrane was gently scratched close to the epiphyseal plate, further destroying its structure and disrupting the balance. Third, saline was used to wash the capsule with sterile water for injection. Finally, we used double needles for percutaneous puncture and lavage to ensure completion lavage. All these initiatives are aimed at treating the non-scraped part of the cyst membrane (adjacent to the epiphyseal plate). Compared with complete excision, these treatments are insufficient, but the combination of multiple methods leads to very satisfactory clinical outcomes.

The percutaneous method is attractive and can be used with materials such as bone marrow or bone substitutes and can optimize results. Many conflicting results have been obtained in different studies regarding the injection of osteoinductive material for the treatment of UBC. Canavese et al. [34] compared the outcomes of percutaneous curettage, intracystic injection of methylprednisolone, and bone marrow for UBC. The results suggest that mechanical disruption of the cyst membrane may be helpful for cyst healing and that this technique may be preferred to simple intracystic injections. Hou et al. [30] retrospectively analyzed the effects of percutaneous curettage, calcium sulfate bone transplantation, and screw drainage with other minimally invasive treatments for UBC, which suggest that medullary decompression plays a special role in the treatment of bone cysts. Sung et al. [31] found that the failure rate of bone marrow aspirate in the treatment of UBC was more than 50% after a single procedure, compared with traditional steroid injection and curettage. Mavcic et al. [35] found that patients with drainage screw had the highest UBC recurrence rate, suggesting that decompression did not cure UBC well. Cho et al. [3] found that although the overall success rates of steroid injection and autologous bone marrow transplantation in the treatment of UBC were similar, the steroid group had higher recurrence after a single procedure and required more injections to achieve healing. In several previous studies, we found that the recurrence rate of intramedullary decompression and drainage group was high, and the autogenous bone marrow transplantation group could not heal completely. The bone graft or not did not affect cyst healing, and cyst size was not correlated with recurrence. This means that UBC is the result of multiple etiological factors. Our treatment is a combined application. The combination of several methods for the treatment of AUBC is also multifaceted based on the etiology of UBC.

UBC consist of benign self-healing fluid-filled tumor-like lesions that can self-heal. Radical excision of the cyst reduces the recurrence rate but increases the morbidity and complication rates. Our experience showed that treatment does not provide thorough clearance, but provides sufficient clearance and promotes sufficient healing. Previous minimally invasive treatments have achieved good results in latent stage UBC, but the curative effect of AUBC is not satisfactory because treatment of the adjacent epiphyseal cyst membrane by minimally invasive operation is insufficient. For sufficient treatment of the cyst membrane, steroids were used to treat the cyst cavity first, followed by percutaneous curettage of the tissue of cystic cyst membrane. Even scraping off the cyst membrane adjacent to the epiphysis ensures adequate safety. We injected sterile water instead of saline for the same purpose. Our combination method demonstrated a satisfactory effect. The operation was minimally invasive, and the patient recovered quickly. All AUBC were well controlled and easily cured. There was no complication.

Our retrospective clinical study has some limitations. We first treated the cavity with steroids to prevent the leakage of methylprednisolone into the medullary cavity. To make full use of methylprednisolone, we keep it in the cavity for 15 min, followed by sterile water injection and percutaneous curettage of the cyst membrane. These procedures greatly increase the operation time. At the same time, curettage of the adjacent epiphyseal cyst membrane also requires experience, and large amount of scraping will injure the epiphysis, causing a growth deformity. Therefore, such cases require more operation time and richer clinical experience.

Pathological fracture is another problem in minimally invasive treatment. Patients who developed pathological fractures pre-hospitalization in our study were treated with plaster external fixation for 1–3 months until fracture healing was achieved. The pathological fracture of AUBC can be healed by external fixation, but most of the cysts cannot be healed, so the cyst still requires treatment. Other limitations of our study include its retrospective nature and the absence of a direct comparative group or randomized comparison. Also, some patients had a shorter radiographic than clinical follow-up and the films were not subjected to blinded review. Our study findings suggest that the treatment of the cyst membrane next to the epiphysis may be key to reducing the recurrence of AUBC.

## Conclusion

For AUBC, minimally invasive treatment is feasible to control cyst progression and then cure it without sequelae. Intracystic methylprednisolone injection, percutaneous curettage, and autogenous bone marrow grafting are an excellent choice of minimally invasive surgery, with a high healing rate, being minimally invasive, and rarely resulting in skeletal malformations.

## Data Availability

All the data used in the article can be obtained from the medical record information system of Xiangya Hospital, Central South University. Any questions or enquiries regarding the present study can be directed to Wei Luo, MD (luowei0928@126.com), as the corresponding author.

## Abbreviations

AUBC: Active unicameral bone cysts
MRI: Magnetic resonance imaging
UBC: Unicameral bone cysts
